# The impact of social support on academic burnout among medical students: the mediating effects of anxiety and depression, and the role of urban-rural differences

**DOI:** 10.3389/fpsyt.2026.1794366

**Published:** 2026-04-01

**Authors:** Hualing Li, Min Liu, Mengxiao Yang, Enze Gao, Xiuyin Gao

**Affiliations:** 1 School of Public Health, Xuzhou Medical University, Xuzhou, Jiangsu, China; 2 Network Ideological and Political Education Center, Xuzhou Medical University, Xuzhou, Jiangsu, China; 3 Department of Community and Health Education, School of Public Health, Xuzhou Medical University, Xuzhou, China; 4 School of Anesthesiology, Xuzhou Medical University, Xuzhou, China; 5 Discipline Inspection Commission, Xuzhou Medical University, Xuzhou, Jiangsu, China

**Keywords:** academic burnout, anxiety, depression, social support, structural equation model

## Abstract

**Purpose:**

An increasing amount of evidence suggests that the prevalence of academic burnout among adolescents is on the rise, especially among high-pressure student groups such as medical students. This study examined the protective effect of social support on academic burnout, the mediating impacts of anxiety and depression in this relationship, and the role of urban-rural differences from medical students.

**Methods:**

We conducted a cross-sectional study, including 1883 medical students. Data were collected using validated self-report questionnaires assessing social support, anxiety, depression, and academic burnout. Perform Pearson correlation analysis and structural equation modeling to examine the associations between social support, anxiety, depression, and academic burnout. And group difference analysis was used in structural equation modeling to explore the impact of urban-rural differences on these relationships.

**Results:**

Correlation analysis revealed a significant negative correlation between social support and academic burnout (r=-0.352, *p* < 0.001). And anxiety and depression showed positive correlations with academic burnout (r=0.509, *p* < 0.001 and r=0.569, *p* < 0.001, respectively). The mediation model indicated that social support had a significant negative impact on academic burnout (*β=*-0.174, 95%CI=-0.215, -0.134). Depression partially mediated the relationship between social support and academic burnout (*β=*-0.153, 95%CI=-0.194, -0.125). Group difference analysis showed that urban-rural differences significantly influenced the relationship between social support and depression (CRD = 2.151, *p* < 0.05).

**Conclusion:**

Social support negatively predicted academic burnout, with depression playing a mediating role. Urban-rural differences had a significant impact on the relationship between social support and depression. Intervention measures should focus on improving social support level and regulating depressive symptoms, and adopting targeted approaches for urban and rural medical students.

## 1 Introduction

Academic burnout refers to a psychological state of fatigue caused by prolonged academic pressure, manifested as emotional exhaustion, a cynical attitude, and a sense of low academic efficiency (1, 2). The term academic burnout originates from occupational burnout and is used to evaluate chronic fatigue in the workplace (3). The conceptual framework of job burnout has been extended to the educational context, given the analogous demands and chronic stressors shared between academic and professional environments. In recent years, academic burnout has become a global issue troubling college students (2, 4). According to a systematic evaluation report (5), moderate levels of burnout syndrome were prevalent among college students from different professions worldwide. In China, the incidence of academic burnout has also been increasing among university students in recent years (6), triggering a widespread concern.

Research on academic burnout among college students has mostly focused on medical student (6, 7). Compared with students in other academic fields, medical students face special psychological pressure and social challenges after completing relatively many and difficult basic courses and theoretical practices (8, 9). Multiple studies have shown that the incidence of academic burnout among medical students is higher than that of their peers (10–12). Notably, researches have confirmed the high incidence of academic burnout among medical students in Jiangsu Province (13, 14), indicating that academic burnout has become a prominent public health issue among medical students in the region. Meanwhile, research have found that the strong demand for medical education and emotional burden have created a toxic environment, making academic burnout common among medical students and having a significant negative impact on their academic performance and mental health (15, 16). Medical students with higher rates of burnout often experience more severe psychological distress, such as anxiety and depression (17–19). Research has found that the strong demand for medical education and emotional burden have created a stressful learning environment, making academic burnout common among medical students and having a significant negative impact on their academic performance and mental health (13, 14). Medical students with higher levels of burnout often experience more severe psychological distress, such as anxiety and depression (15–17). Given the high prevalence and adverse consequences of academic burnout among medical students in Jiangsu Province, identifying modifiable influencing factors and developing targeted intervention strategies is particularly urgent and meaningful for local medical education and healthcare talent cultivation.

So far, some studies on academic burnout among medical students have mainly focused on demographic and environmental factors that influence academic burnout (16, 20, 21), while others primarily used epidemiological analysis methods such as systematic reviews (5, 6). Only a few studies have considered the joint mechanism of multiple variables in academic burnout. Given the high prevalence of academic burnout and its significant impact on students’ mental health and academic performance, it is important to further explore the joint influence mechanism of more factors in academic burnout.

### 1.1 Social support and academic burnout

Social support is defined as an individual’s emotional perception and satisfactory evaluation of being respected, supported, and understood in society (22). It mainly includes support from family, friends, and significant others, which can promote individual’s psychological and physical well-being through emotional listening, empathetic understanding, and practical assistance (23). In contrast, insufficient social support may increase vulnerability to negative psychological outcomes (24). According to the stress buffering model (25), social support acts as a protective factor that alleviates the negative impact of external environment on individuals. A study exploring the risk and protective factors of academic burnout among college students revealed a negative correlation between social support and academic burnout, and positively predicting that social support can effectively reduce levels of burnout, indicating that high social support effectively reduces burnout levels (26). A growing body of empirical evidence further confirms the close correlation between social support and academic burnout (26, 27), and emphasizes the crucial protective role of social support against academic burnout. Therefore, we propose the first hypothesis (H1): social support can directly reduce academic burnout among medical students.

### 1.2 Anxiety as a mediator

Anxiety is a common negative emotional response (28), which is an internal psychological reflection of an individual’s experience of external pressure and threats. When anxiety becomes persistent or excessive, it can significantly impair mental health and daily function. The theory of anxiety buffer disruption suggests that the failure of psychological buffering mechanisms may trigger heightened anxiety (25), further resulting in maladaptive outcomes such as increased fatigue and burnout. If such negative emotional states are not effectively alleviated, they may gradually develop into academic burnout (29). The theoretical perspective indicates that anxiety serves as a key risk factor for academic burnout. In addition, previous researches have confirmed that social support is negatively related to anxiety, and high-level social support can alleviate anxiety and other negative emotions (30, 31). Therefore, we propose the second hypothesis (H2): anxiety can mediate the impact of social support on academic burnout among medical students.

### 1.3 Depression as a mediator

Depression is characterized by low enthusiasm, hopelessness, inferiority, and diminished motivation, typically manifested as a lack of interest in daily activities (32). Similar to anxiety, depression is recognized as another important risk factor for academic burnout. According to the stress generation hypothesis (33), depressive symptoms can induce negative cognition and emotions, which affect individuals’ behaviors and eventually lead to academic burnout. And a positive association between depression and academic burnout has been consistently demonstrated (34), with higher depression symptoms often experiencing higher levels of academic burnout. Simultaneously, social cognitive theory points out that individual behavior is shaped by external environment and internal psychological processes (35). Ample evidence has also confirmed the close relationship between social support and depression (30, 36), leading us to propose the third hypothesis (H3): depression can mediate the impact of social support on academic burnout among medical students.

### 1.4 Group difference by urban-rural

Crucially, the strength of the proposed pathways, including the direct effect of social support on academic burnout and the indirect effects via anxiety and depression, may not be identical across different subgroups. Previous research examing urban-rural differences in social support have showed inconsistent results. For example, a study conducted in Xiamen, China (37), suggested that rural students had higher and more stable level of social support than urban students. In contrast, the opposite conclusion found that rural adolescents perceive limited emotional and material support from family and external sources (38), resulting in relatively low levels of social support. Similarly, mixed findings exist regarding urban-rural differences in academic burnout. Western researches found that rural environment often bring unique sources of stress (39), which may contribute to a higher incidence of academic burnout among rural students. However, limited attention has been paid to urban-rural differences in academic burnout among students in China.

The inconsistent findings highlight the necessity to further explore the moderating role of urban-rural students in Chinese unique sociocultural context. Compared with urban students, rural students often face disparities in educational resources, economic conditions, and social environments (40, 41). These differences may shape their psychological experiences and influence how social support affects anxiety, depression, and further academic burnout. Therefore, urban-rural backgrounds may act as important moderators in the relationship between social support and academic burnout. Accordingly, we propose the fourth hypothesis (H4): the direct and indirect pathways from social support to academic burnout may differ between urban and rural medical students.

### 1.5 Present study

**Figure 1:**
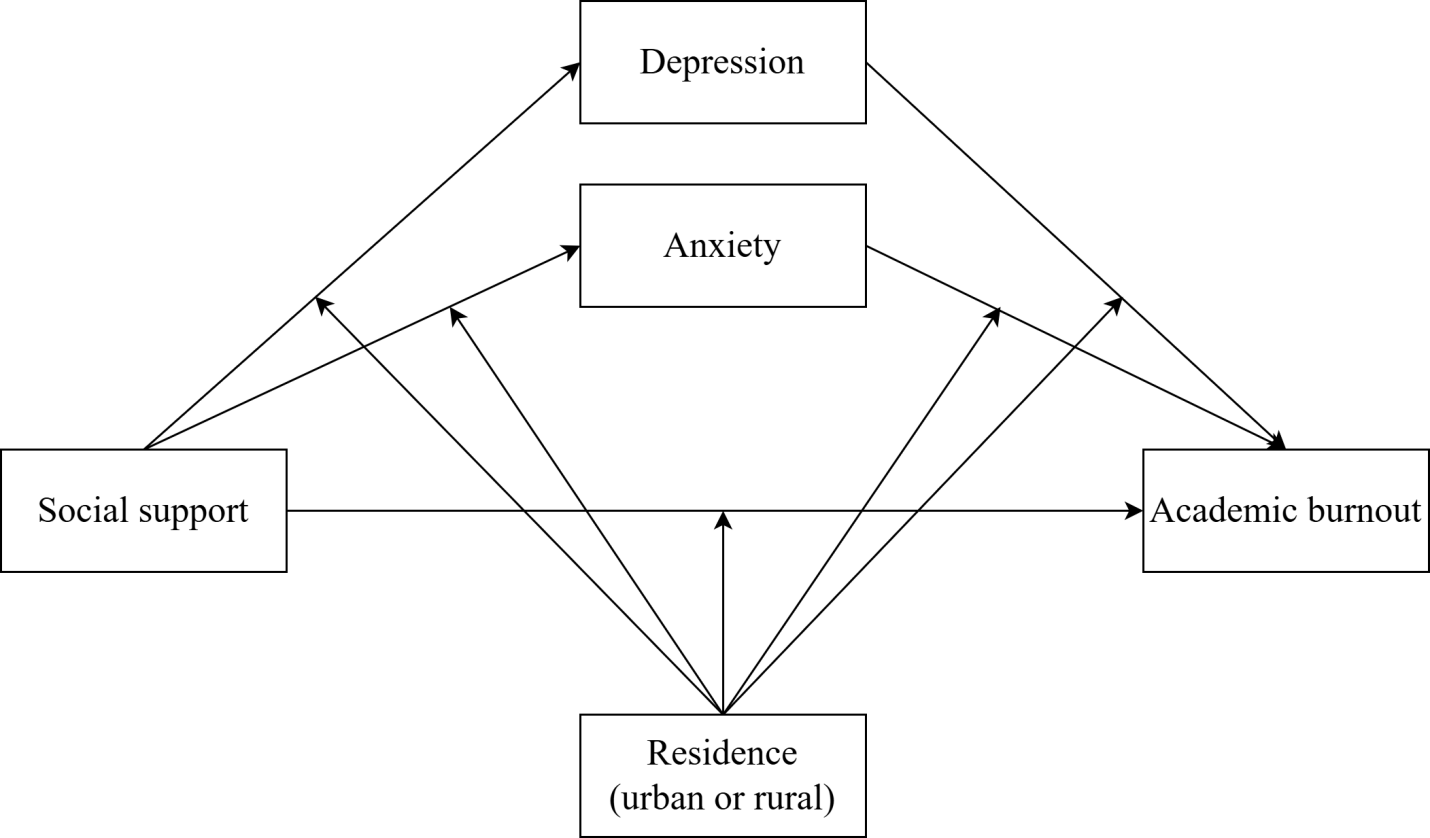
Conceptual framework.

As mentioned above, we hypothesized a mediation model as shown in **Figure 1**, we aimed at exploring the protective effect of social support on academic burnout and the mediating role of anxiety and depression in this relationship, as well as investigating the impact of urban-rural differences on the relationship between variables. Specifically, this study attempts to test the following four hypotheses:

H1: social support can directly reduce academic burnout among medical students.
H2: anxiety can mediate the impact of social support on academic burnout among medical students.
H3: depression can mediate the impact of social support on academic burnout among medical students.
H4: the direct and indirect pathways from social support to academic burnout may differ between urban and rural medical students.

## 2 Methods

### 2.1 Participants

Participants were recruited using convenience sampling from two medical universities in Jiangsu Province, China: Nanjing Medical University and Xuzhou Medical University. The survey was conducted online from March 2024 to May 2024 via “wenjuanxing”, a professional online questionnaire platform, and the questionnaire link was distributed through the social media application WeChat. A total of 2,072 questionnaires were distributed, and 1,883 valid responses were retained after excluding those that were incomplete or inconsistent, with an effective response rate of 90.88%. This study was approved by the Ethics Committee of Xuzhou Medical University and conducted in accordance with the Helsinki Declaration.

Inclusion criteria: undergraduate medical students from freshman to fifth year who voluntarily participated after reading the study instructions. Exclusion criteria (1): The questionnaire completion time less than two minutes; (2) those who have obvious contradictions in answering questions; (3) those who choose the same answer for all options.

### 2.2 Measures

#### 2.2.1 Social demographic characteristics

The study collected information on social demographic characteristics, including gender, grade, only child, residence, ethnic group, monthly living expenses (yuan), father’s education level, and mother’s education level. Residence, as a fundamental demographic variable, was included in the study as a categorical variable to distinguish between urban and rural students (1=urban, 2=rural).

#### 2.2.2 Social support

The social support level of medical students was measured using the Perceived Social Support Scale (PSSS-12) (22). The Chinese version of the scale was translated by Huang et al. and its effectiveness was verified (42). The scale consists of 12 items, including three dimensions: family support (4 items), friend support (4 items), and other support (4 items). It utilizes a 7-point Likert rating scale ranging from 1 (strongly disagree) to 7 (strongly agree). The higher the total score, the higher the individual’s level of social support. The internal consistency of the scale in this study was good, with a Cronbach’s α coefficient of 0.975.

#### 2.2.3 Academic burnout

Academic burnout was assessed using the Adolescent Student Burnout Inventory (ASBI) developed by Wu Yan (43). This scale consists of 16 items rated with a 5-point Likert scale ranging from 1 (strongly disagree) to 5 (strongly agree). The higher scores represent the higher levels of learning burnout. This scale has demonstrated reliable reliability and validity in previous studies (44). The Cronbach’s alpha of this scale in this study was 0.951.

#### 2.2.4 Anxiety symptoms

The Chinese version of the General Anxiety Disorder-7 scale (GAD-7) was used to measure medical students’ anxiety symptoms (44). The GAD-7 consists of seven items and each item is scored using the Likert-4 rating scale ranging from 0 (not at all) to 3 (almost every day), and the total score ranges from 0 to 21, with the cut-off values of 5, 10, and 15 indicating mild, moderate, and severe anxiety symptoms, respectively. GAD-7 has been proven to be a reliable and valid measure of anxiety in the Chinese population (45, 46). The Cronbach’s alpha of this scale in this study was 0.956.

#### 2.2.5 Depression symptoms

The present study used the Patient Health Questionnaire-9 (PHQ-9) to assess the depression symptom among medical students (47). The scale consists of nine items that measure the frequency of depressive symptoms occurring in the past two weeks. PHQ-9 uses a 4-point Likert scale and the total score ranges from 0 to 27, with critical values of 5, 10, and 15 representing mild, moderate, and severe depressive symptoms, respectively. The PHQ-9 scale had good reliability and validity in the Chinese population (48), and with a Cronbach’s alpha of 0.943 in this study.

## 3 Analytical strategies

### 3.1 Descriptive analysis

All data were analyzed using SPSS 24.0 and Amos 24.0, with *p <* 0.05 considered statistically significant. Quantitative data are presented as mean ± standard deviation, and Count data is described in frequency and percentage. T-tests and Analysis of Variance (ANOVA) were conducted to explore the differences between social demographic characteristics and key variables.

### 3.2 Pearson correlation analysis and structural equation modeling

Pearson correlation analysis was used to clarify the correlations between social support, anxiety, depression and academic burnout. Structural equation modeling was established using Amos 24.0 software, and the maximum likelihood estimation method was employed to assess the hypothesized model. In this study, we evaluated the fit of the model through path analysis (49). In path analysis, we calculated a series of indicators, including the chi-square to degrees of freedom ratio (CMIN/DF), goodness-of-fit index (GFI), adjusted goodness-of-fit index(AGFI), comparative fit index (CFI), incremental fit index (IFI), standardized root-mean-square residuals (SRMR), and root-mean-square error of approximation (RMSEA). A model is considered to have a satisfactory fit if it shows indices above 0.90 for GFI, AGFI, CFI and IFI, and below 0.08 for SRMR and RMSEA (50, 51). Bootstrap analysis was conducted to test the total, indirect, and direct effects of the hypothesized model. Indirect effects were considered statistically significant if their 95% confidence intervals(CI) did not contain zero. Multi group structural equation model was used to evaluate the urban-rural differences in the whole model, and the critical difference ratio (CRD) was used to compare the structural path coefficient of each group. If the absolute value of CRD is higher than 1.96, there is a significant inter-group difference at the level of *p* < 0.05.

## 4 Results

### 4.1 Common method biases test

To evaluate the potential influence of common method biases in cross-sectional design, we conducted Harman’s single-factor test. The results indicated that through principal component factor analysis, we extracted 6 feature values greater than 1, explaining 72.973% of the total variance. The variance explained by the first factor is 38.481%, which is below the critical threshold of 40%, indicating that common method bias is not a serious issue in this study.

### 4.2 Preliminary analysis

**Table 1** displays group differences in participants’ social demographic characteristics and key variables. In this study, there were 885 urban students and 998 rural students. There was no statistical significance in the gender (*χ^2^* = 1.352, *p* = 0.245), nationality (*χ^2^* = 0.029, *p* = 0.866) between urban and rural students, but there was significance in other demographic variables (all *p* < 0.001). In the analysis of key variables, compared with the rural students, the urban students showed a higher level of social support (F = 19.085, *p* < 0.001), and the group differences in other key variables were not statistically significant (all *p*>0.05).

**Table 1:** Group differences in characteristics and key variables among participants.

Variables	N(%)/M(SD)	Residence		*χ^2^/*F	*p*
		Urban (n=885) N(%)/M(SD)	Rural (n=998) N(%)/M(SD)		
Social demographic variables					
Gender				1.352	0.245
Male	780(41.42)	379(20.13)	401(21.30)		
Female	1103(58.58)	506(26.87)	592(31.71)		
Grade				25.009	<0.001
Freshman	684(36.33)	278(14.76)	406(36.33)		
Sophomore	379(20.13)	171(9.08)	208(11.05)		
Junior	438(23.26)	233(12.37)	205(10.89)		
Senior	294(15.61)	160(8.50)	134(7.12)		
Fifth year	88(4.67)	43(2.28)	45(2.39)		
Only child				303.512	<0.001
Yes	793(42.11)	559(29.69)	234(12.43)		
No	1090(57.89)	326(17.31)	764(40.57)		
Ethnic group				0.029	0.866
The Han ethnic group	1807(95.96)	850(45.14)	957(50.82)		
Other ethnic group	76(4.04)	35(1.86)	41(2.18)		
Monthly living expenses (yuan)				96.965	<0.001
<1000	132(7.01)	35(1.86)	97(5.15)		
1001-2000	1309(69.52)	560(29.74)	749(39.78)		
2001-3000	377(20.02)	240(12.75)	137(7.28)		
>3000	65(3.45)	50(2.66)	15(0.80)		
Father’s level of education				439.083	<0.001
Junior high school or below	735(39.03)	169(8.98)	566(30.06)		
Senior or vocational school	599(31.81)	267(14.18)	332(17.63)		
Undergraduate	498(26.45)	404(21.46)	94(4.99)		
Master or doctor	51(2.71)	45(2.39)	6(0.32)		
Mother’s level of education				538.694	<0.001
Junior high school or below	889(47.21)	187(9.93)	702(37.28)		
Senior or vocational school	543(28.84)	310(16.46)	233(12.37)		
Undergraduate	407(21.61)	351(18.64)	56(2.94)		
Master or doctor	44(2.34)	37(1.97)	7(0.37)		
Key variables					
Academic burnout	2.31(1.29)	2.26(1.36)	2.36(1.21)	2.564	0.109
Social support	5.21(1.27)	5.35(1.30)	5.09(1.23)	19.085	<0.001
Anxiety	0.52(0.67)	0.51(0.71)	0.52(0.63)	0.115	0.735
Depression	0.57(0.62)	0.56(0.65)	0.58(0.58)	0.213	0.644

### 4.3 Correlation between variables

**Table 2** presents the social support, anxiety and depression with academic burnout. There was a negative correlation between academic burnout and social support (r=-0.352, *p* < 0.001), a positive correlation between academic burnout and anxiety (r=0.509, *p* < 0.001), and a positive correlation between academic burnout and depression (r=0.569, *p* < 0.001).

**Table 2:** Correlation between academic burnout, social support, anxiety and depression.

Variables	$\bar{X}$ ± *SD*	1	2	3	4
1Academic burnout	2.31 ± 1.29	1			
2Social support	5.21 ± 1.27	-0.352***	1		
3Anxiety	0.52 ± 0.67	0.509***	-0.250***	1	
4Depression	0.57 ± 0.62	0.569***	-0.264***	0.821***	1

***indicates *p* < 0.001.

### 4.4 Analysis of mediating effects

**Figure 2:**
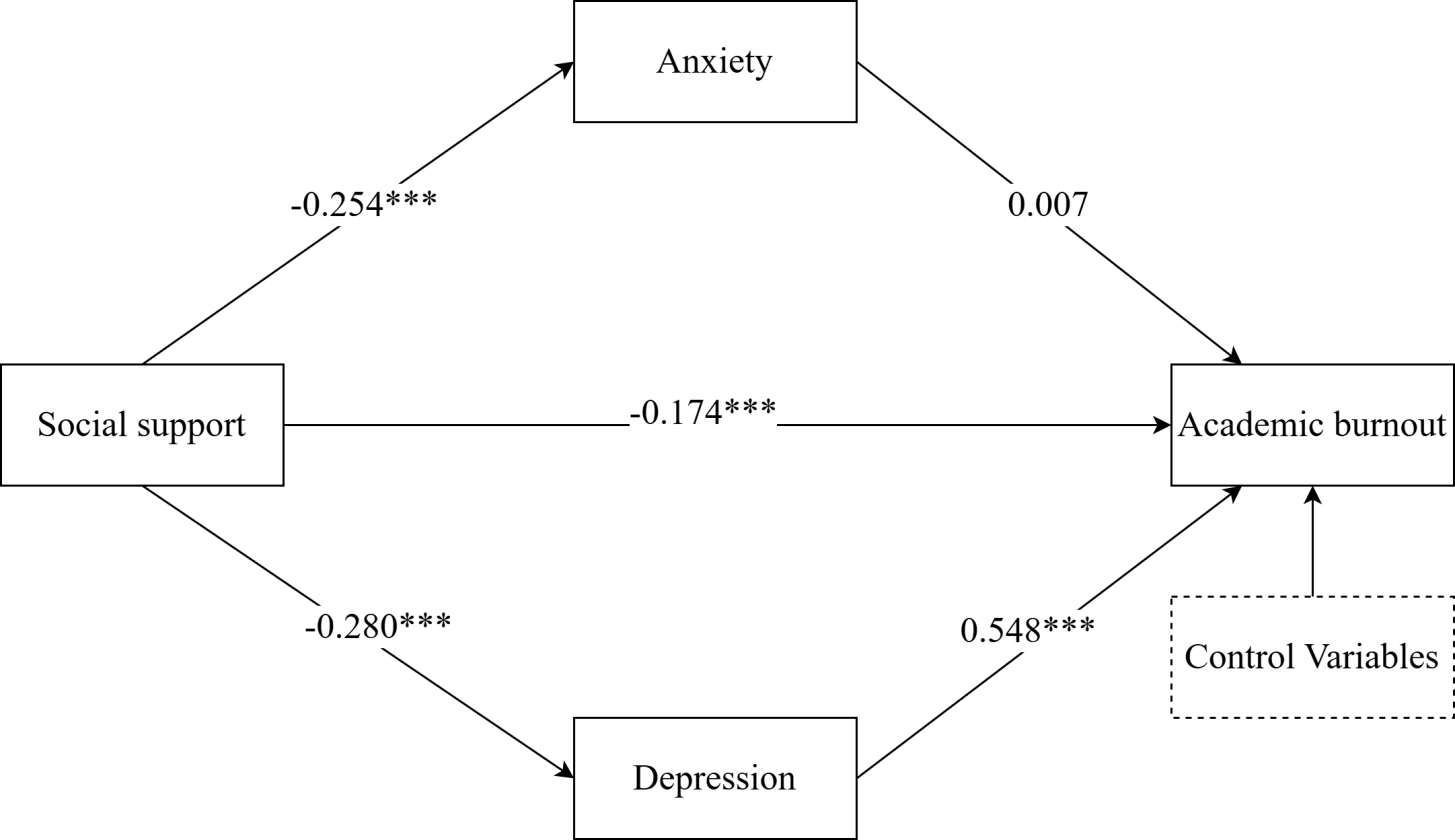
A mediation model of the relationship between social support and academic burnout through anxiety and depression (full sample). Control variables include gender, grade, ethnicity, father’s level of education, mother’s level of education. The values in the figure are standardized path coefficients, *** indicates p < 0.001.

Structural equation modeling was constructed to explore the association between social support and academic burnout, with anxiety and depression as two parallel mediating roles. The initial model results showed unsatisfactory fit. Further modifications were made to the model by establishing correlations among the residuals of social support, academic burnout, anxiety and depression dimensions. Revised model results showed that all fitting indices met the standard requirements, as shown in **Table 3**. Standardized path coefficients are displayed in **Figure 2**.

**Table 3:** Fitness indexes of the model (full sample).

Variables	CMIN/DF	GFI	AGFI	IFI	CFI	RMSEA	SRMR
Reference	<5.0	>0.9	>0.9	>0.9	>0.9	<0.08	<0.08
Initial	17.153	0.653	0.619	0.823	0.823	0.093	0.165
Revised	4.167	0.917	0.904	0.967	0.967	0.041	0.049

CMIN/DF=degrees of freedom, GFI=goodness-of-fit index, AGFI= adjusted goodness-of-fit index, IFI=incremental fit index, CFI= comparative fit index, SRMR=standardized root-mean-square-residual, RMSEA=root-mean-square-error-of- approximation.

We assessed the mediating effects using the bias-corrected percentile bootstrap method, and the results for the total, direct, and indirect effects are presented in **Table 4**. Social support directly and negatively predicted academic burnout (*β=*-0.174, 95%CI=-0.215, -0.134), accounting for 52.9% of the direct effect, confirming H1. Social support had not a significant impact on academic burnout through anxiety (*β=*-0.002, 95%CI=-0.023, 0.030), not confirming H2. Social support had a significant impact on academic burnout through depression (*β=*-0.153, 95%CI=-0.194, -0.125), with a indirect effect of 46.6%, confirming H3.

**Table 4:** The standardized total, direct, and indirect effects of social support on academic burnout with anxiety and depression as mediators.

Model path	Full sample			Urban			Rural		
	*β*	95%CI	Percent (%)	*β*	95%CI	Percent (%)	*β*	95%CI	Percent (%)
Total effect	-0.330***	(-0.370,-0.278)	100	-0.369***	(-0.440,-0.301)	100	-0.282***	(-.0345,-0.203)	100
Direct effect									
SS→AB	-0.174***	(-0.215,-0.134)	52.9	-0.174***	(-0.224,-0.107)	47.1	-0.168***	(-0.230,-0.102)	59.6
Indirect effect									
SS→Anxiety→AB	-0.002	(-0.023, 0.030)	0.6	-0.002	(-0.046.0.033)	0.6	-.0.002	(-0.022,0.028)	0.6
SS→Depression→AB	-0.143***	(-0.194,-0.125)	46.6	-0.193***	(-0.263,-0.127)	52.3	-0.112***	(-0.153,-0.083)	39.9

***indicates *p* < 0.001, SE, standard error; CI, confidence interval; SS, social support; AB, academic burnout.

### 4.5 Test of group differences

Multi-group structural equation modeling (SEM) was conducted to test urban-rural differences in path coefficients. Firstly, the invariance of the measurement was verified and the results indicated that the measurement model remained unchanged (*p*>0.05). This result suggested that the factor loadings in the measurement model were equivalent between urban and rural students. Subsequently, we compared the unconstrained model and constraint model of structural path variation, where the factor loadings, covariance, weights, and residuals for urban and rural were set equally in the constraint model. The results showed that the unconstrained model (*χ^2^* = 5360.296, df=1872) and the constrained model (*χ^2^* = 5438.246, df=1919) had a significant difference (*p* < 0.05).

**Figure 3:**
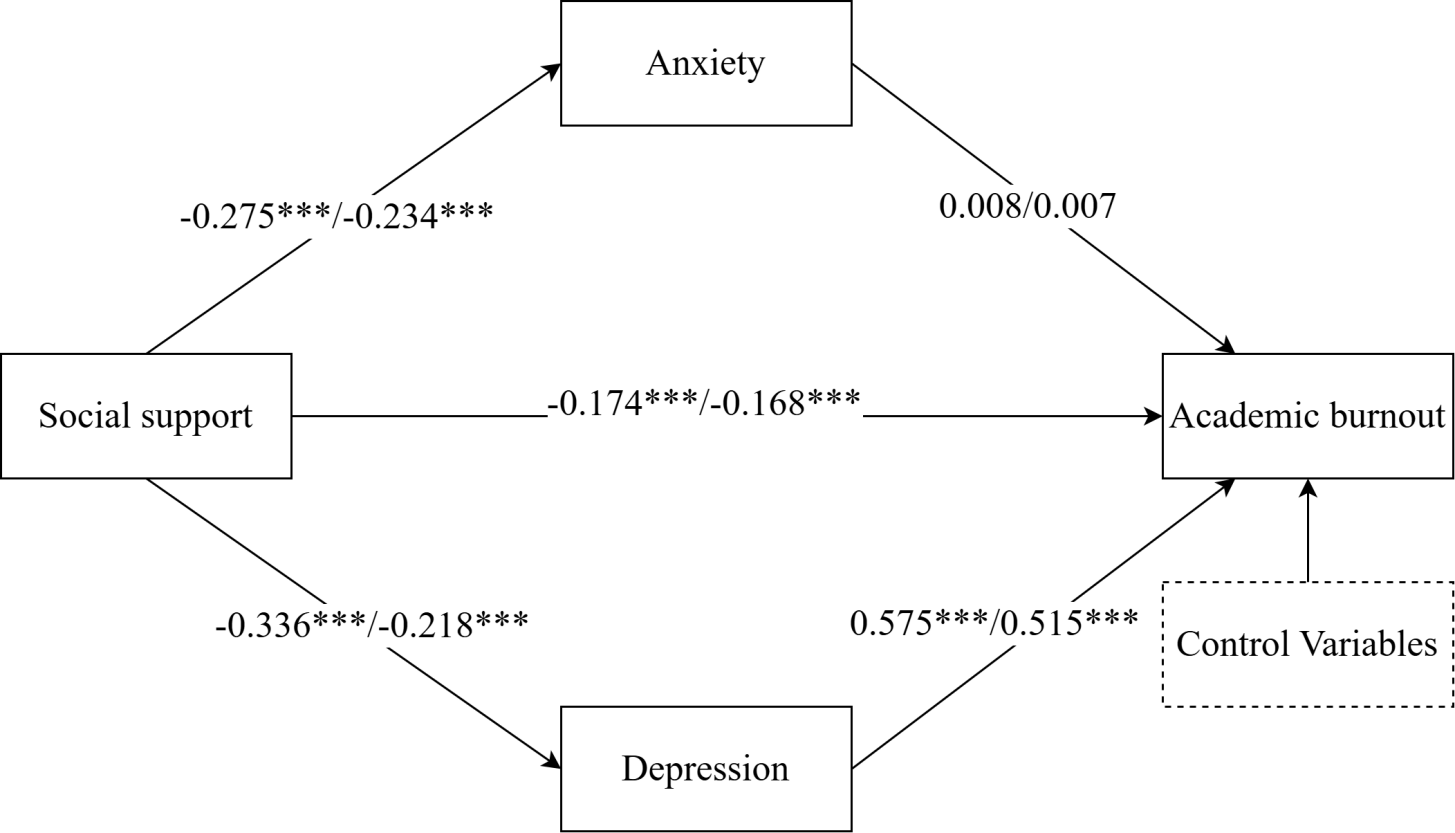
Standardized structural model (grouped by urban-rural). The two values from left to right on each path represent the standardized coefficients corresponding to the urban population model and rural population, respectively. *** indicated *p* < 0.001.

The critical ratios of differences (CRD) testing revealed a significant urban-rural differences in the path from social support to depression (CRD = 2.151, *p* < 0.05), confirming H4. As shown in **Figure 3**, social support had a significant negative impact to depression on both urban and rural medical students, but the effect was stronger in urban medical students (*β* = -0.336, *p* < 0.001) than in rural medical students (*β* = -0.218, *p* < 0.001). No significant urban-rural differences were observed in other structural pathways (all *p* < 0.05).

## 5 Discussion

This study aimed to explore the mediating roles of anxiety and depression in the relationship between social support and academic burnout among medical students, and the impact of urban-rural differences on this relationship. The results indicated that social support not only directly affected academic burnout, but also indirectly affected academic burnout through depression. Moreover, there were significant differences between urban and rural medical students in the path from social support to depression.

### 5.1 Direct effect of social support on academic burnout

The study showed that social support was negatively correlated with academic burnout among medical students. This finding was consistent with previous researches (52, 53). According to the social support buffer hypothesis (54), social support functions as a protective factor that reduces individuals’ cognitive of pressure and threat. Medical university is characterized by intensive work, heavy academic load and pressure, which render medical students vulnerable to academic burnout. However, it is worth noting that in the context of medical education, social support from teachers, academic mentors cannot be ignored. Medical teachers and mentors directly guide professional development and shape clinical abilities. They can be both a source of support and a source of stress, and their influence on the psychology of medical students far exceeds that of families or peers. Sufficient support can help medical students cope with academic and clinical challenges, and perceive academic pressure in a more positive and optimistic way. Multiple researches exploring the relationship between social support, resilience, and burnout have found that (14, 55, 56), medical students who receive more social support naturally have stronger resistance to stress, helping them reduce the occurrence of academic burnout. Our study further confirms that improving social support can represent a feasible and effective strategy to prevent or reduce academic burnout among medical students.

### 5.2 Mediating roles of anxiety and depression

An unexpected finding was that anxiety did not mediate the relationship between social support and academic burnout among medical students. Although social support negatively predicted anxiety, but anxiety failed to significantly predict academic burnout, which was inconsistent with many previous researched results (57, 58). We first considered that this may be related to the multidimensional structure of academic burnout. Generally speaking, academic burnout includes three dimensions: emotional exhaustion, cynicism, and low personal achievement. Research has found that the strongest link exists between anxiety and emotional exhaustion in burnout, while with weaker predictive effects on cynicism and low personal achievement (59). Secondly, we believe that the irrelevant result in this study may be related to the academic burnout scale we used. We found that over half of studies using MBI testing had a lower impact on the relationship between anxiety and burnout compared to studies using other burnout measurement scales (60). Finally, we also considered whether medical students are generally in high-pressure environments with overall high levels of anxiety, and individual differences may be masked by group averages, leading to a decrease in the predictive power of anxiety on their academic burnout.

On the contrary, the results showed that depression played a significant mediating role between social support and academic burnout. Social support negatively predicted depression, aligning with previous research findings (61–63). As the buffering hypothesis suggests (54), social support enhances individuals’ ability to cope with stressful events, and alleviates the negative impact of emotions such as depression. Meanwhile, depression can significantly affect academic burnout, which also confirmed previous research (64, 65). According to the stress affective disorder hypothesis (66), prolonged exposure to stress can lead to difficulties in facing and regulating emotion, forming a vicious cycle of “stress affective disorder psychological illness”, which has been shown to lead to harmful consequences such as academic burnout (67). Therefore, we believe that finding ways to alleviate depression is another effective intervention to reduce academic burnout among medical students.

### 5.3 Urban-rural differences on academic burnout

Our results revealed that the effect of social support on depression was significantly stronger among urban students than rural students. This finding may be explained from the perspective of social capital theory (68). Rural areas usually present strong bonding social capital characterized by close family and community ties. In contrast, urban contexts provide more abundant and accessible bridging social capital, such as diverse social networks, formal support systems, and extended social resources. The stronger predictive effect of social support on depression among urban students may be mainly driven by bridging social capital, which helps urban students obtain diversified support more effectively. According to the stress buffering model, social support can buffer the negative impact of stress events (54). Urban students with more access to bridging social capital may received more effective social support than rural students, thus showing stronger stress resistance and depression relief of them.

This finding adds a new dimension to interventions for academic burnout, emphasizing the importance of considering urban-rural backgrounds when developing targeted interventions aimed at reducing academic burnout. For rural medical students, intervention measures should focus on expanding social resources and building bridging social social capital. Specifically, medical universities can provide more counseling services, peer support groups, and online psychological support platforms to compensate for the relatively limited offline social resources in rural contexts. While for urban medical students, interventions should focus on utilizing the existing social support structures to continuously enhance the quality and depth of social support of them, including strengthening communication between students and teachers, improving the quality of support, and guiding students to make better use of diverse social resources to alleviate depression and buffer academic stress, in order to maximize the effectiveness of social support.

## 6 Limitations

Firstly, as a cross-sectional study, this study cannot explore the long-term effects and causal relationships between variables. Secondly, convenience sampling was used for participant recruitment, which may reduce the representativeness of the sample and limit the generalizability of the present findings to other medical students outside Jiangsu Province. Thirdly, in addition to anxiety and depression, other potential mediating roles that may explain the mechanism between social support and academic burnout warrant further exploration, such as resilience or self-efficacy. Finally, we did not consider the long-term impact of the COVID-19 pandemic, which may have permanently changed the social support structure, such as increasing reliance on digital and online communication. Such changes may influence how social support is perceived and obtained, which in turn affects anxiety, depression and academic burnout among medical students. These factors should be fully considered in future research.

## 7 Conclusion

This study examined the roles of anxiety and depression in the relationship between social support and academic burnout among medical students, found that depression partially mediated between these two variables, and there were significant differences in the pathway of social support and depression among urban and rural students. In the future, intervention measures for medical students’ academic burnout should pay attention to their levels of social support and depression situation, implement different intervention plans for urban and rural students to increase social support, alleviate depression, and ultimately reduce the occurrence of academic burnout.

## Data Availability

The raw data supporting the conclusions of this article will be made available by the authors, without undue reservation.
